# Is bone marrow oedema in patients with labral tear an indicator of hip pain?

**DOI:** 10.1186/s13018-022-03243-w

**Published:** 2022-09-15

**Authors:** Tomohisa Koyama, Kensuke Fukushima, Kentaro Uchida, Yoshihisa Ohashi, Katsufumi Uchiyama, Naonobu Takahira, Masashi Takaso

**Affiliations:** 1 Department of Orthopaedic Surgery, School of Medicine, Kitasato University, 1-15-1, Kitasato, Minami-Ku, Sagamihara city, Kanagawa 252-0374 Japan; 2 Department of Patient Safety and Healthcare Administration, School of Medicine, Kitasato University, 1-15-1, Kitasato, Minami-ku, Sagamihara city, Kanagawa 252-0374 Japan; 3 Department of Rehabilitation, School of Allied Health Sciences, Kitasato University, 1-15-1 Kitasato, Minami-ku, Sagamihara, Kanagawa 252-0374 Japan

**Keywords:** Bone marrow oedema, Labral tear, Modified Harris hip score, Hip pain

## Abstract

**Background:**

Hip labral tear (LT) causes various degrees of hip pain, for which there are few objective measures. Bone marrow oedema (BME), characterized by a diffuse, widely spreading change in the bone marrow, is observed in some patients with LT. However, its pathological role has not been fully understood. The purpose of this study was to investigate the prevalence of BME on hip magnetic resonance imaging (MRI) in patients with LT and to determine whether BME was an objective indicator of hip pain.

**Methods:**

In total, 84 patients with LT who underwent MRI scanning under the same conditions were included. We determined the presence or absence of BME and its size on MRI and evaluated the relationships between BME and sex, age, and pain and total scores on the modified Harris hip score (MHHS). In addition, we collected data on surgical treatments such as hip arthroscopy within a one-year follow-up period and examined whether the presence of BME affected the course of therapy.

**Results:**

BME was found in 34.5% of patients. MHHS pain and total scores were significantly lower in patients with BME (MHHS pain score: non-BME vs. BME ≤ 1 cm: *p* = 0.022, non-BME vs. BME > 1 cm: *p* < 0.001; MHHS total score: non-BME vs. BME ≤ 1 cm: *p* = 0.131, non-BME vs. BME > 1 cm: *p* = 0.027). The presence of BME did not differ between patients who did and did not undergo surgery during follow-up (*p* = 0.563).

**Conclusion:**

BME on MRI in patients with LT might be an indicator of hip pain and hip joint dysfunction.

## Background

Patients with hip labral tear (LT) often experience anterior hip pain and/or groin pain. They also suffer from a variety of mechanical symptoms such as clicking, locking, catching, and giving way [1, 2]. Certain motions such as hyper-abduction, hyper-extension, and external rotation are known to be the cause motions of LT [3–5]. However, most of the cases with LT occur without specific triggering events and with varying degrees of pain [6]. There is no clear objective measure of the degree of pain in LT, and some cases are difficult to treat.

Bone marrow oedema (BME), characterized by a diffuse, widely spreading change in the bone marrow, is a finding on magnetic resonance imaging (MRI). BME shows low signal intensity on T1-weighted images and high signal intensity on T2-weighted and short tau inversion recovery (STIR) images [7–9].

BME has been well known for painful osseous conditions, such as transient osteoporosis [10], osteonecrosis [11], and subchondral insufficiency fracture of the hip [12]. In knee osteoarthritis (OA), BME has been shown to have a high correlation with the severity of pain and articular cartilage disorder [13, 14]. Bone and bone marrow have a high number of nociceptive fibres, suggesting that bone may contribute to the pain profile. It is believed that BME causes accumulation of extracellular fluid in painful osseous conditions, thereby leading to an increase in intramedullary pressure [15]. This may affect signalling from nociceptors and increase pain. Therefore, mechanical reduction in intramedullary pressure, such as core decompression, has been reported to relieve pain in some patients [16].

LT, cartilage loss, and BME have been reported to be interrelated [17]. However, the relationship between hip pain in patients with LT and BME has not been fully understood. Therefore, we aimed to examine the prevalence of BME in patients with LT and determine whether it could be an objective indicator of hip pain.

## Patients and methods

### Patients

Approval for this study was obtained from our Institutional Review Board (B20-276). The need for informed consent was waived because of the retrospective study design.

**Fig. 1:**
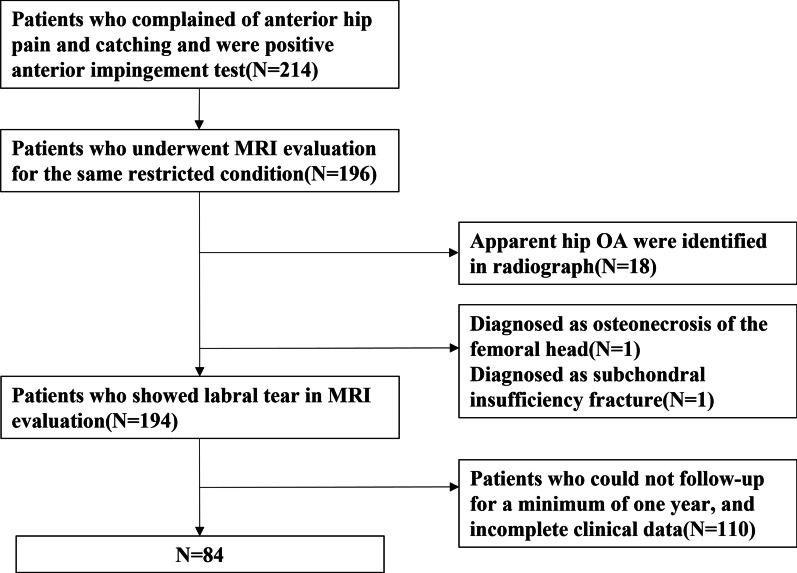
Flow chart of the study. *MRI* Magnetic resonance imaging, *OA* Osteoarthritis

We retrospectively reviewed 84 patients who visited our outpatient clinic from 2014 to 2020 complaining of anterior hip pain and catching, and who were positive for anterior impingement test and underwent MRI evaluation for the same restricted condition resulting in a diagnosis of LT. We excluded patients who showed apparent hip OA in hip radiographs, and those who were diagnosed as having osteonecrosis and subchondral insufficiency fracture. The grade of hip OA was assessed radiographically according to the Kellgren–Lawrence (K–L) classification [18]. The diagnosis of osteonecrosis and subchondral insufficiency fracture in MRI was performed as described by Yamamoto et al. [19]. In addition, the patients who could not follow up for a minimum of one year were excluded. A flow chart demonstrating the inclusion and exclusion process is shown in Fig. 1.

For all patients, data regarding sex, age, and the pain and total scores on the modified Harris hip score (MHHS) at the first presentation were obtained from medical records.

### Assessment of bone marrow oedema

All MR images assessed in this study were investigated under the same conditions. MRI scans were acquired using a MAGNETOM Skyra 3 T system (Siemens Healthineers, Erlangen, Germany) or Discovery MR750w 3 T system (GE Healthcare, Waukesha, WI, USA). The acquisition parameters were as follows: (1) repetition time, 4600 ms; echo time, 64 ms; number of excitations, 2; 15 slices; slice thickness, 3 mm; slice gap, 1 mm; field of view, 160 mm; acquisition matrix, 320 × 240; reconstruction matrix, 640 × 480; and acquisition time, 2 min and 47 s for the MAGNETOM Skyra 3 T system; and (2) repetition time, 3600 ms; echo time, 102 ms; slice excitation, 2, 16 slices; slice thickness, 3 mm; slice gap, 1 mm; field of view, 160 mm; acquisition matrix, 320 × 224; reconstruction matrix, 512 × 512; and acquisition time, 2 min and 17 s for the Discovery MR750w 3 T system. All radiological assessments were independently performed by two observers.

**Fig. 2:**
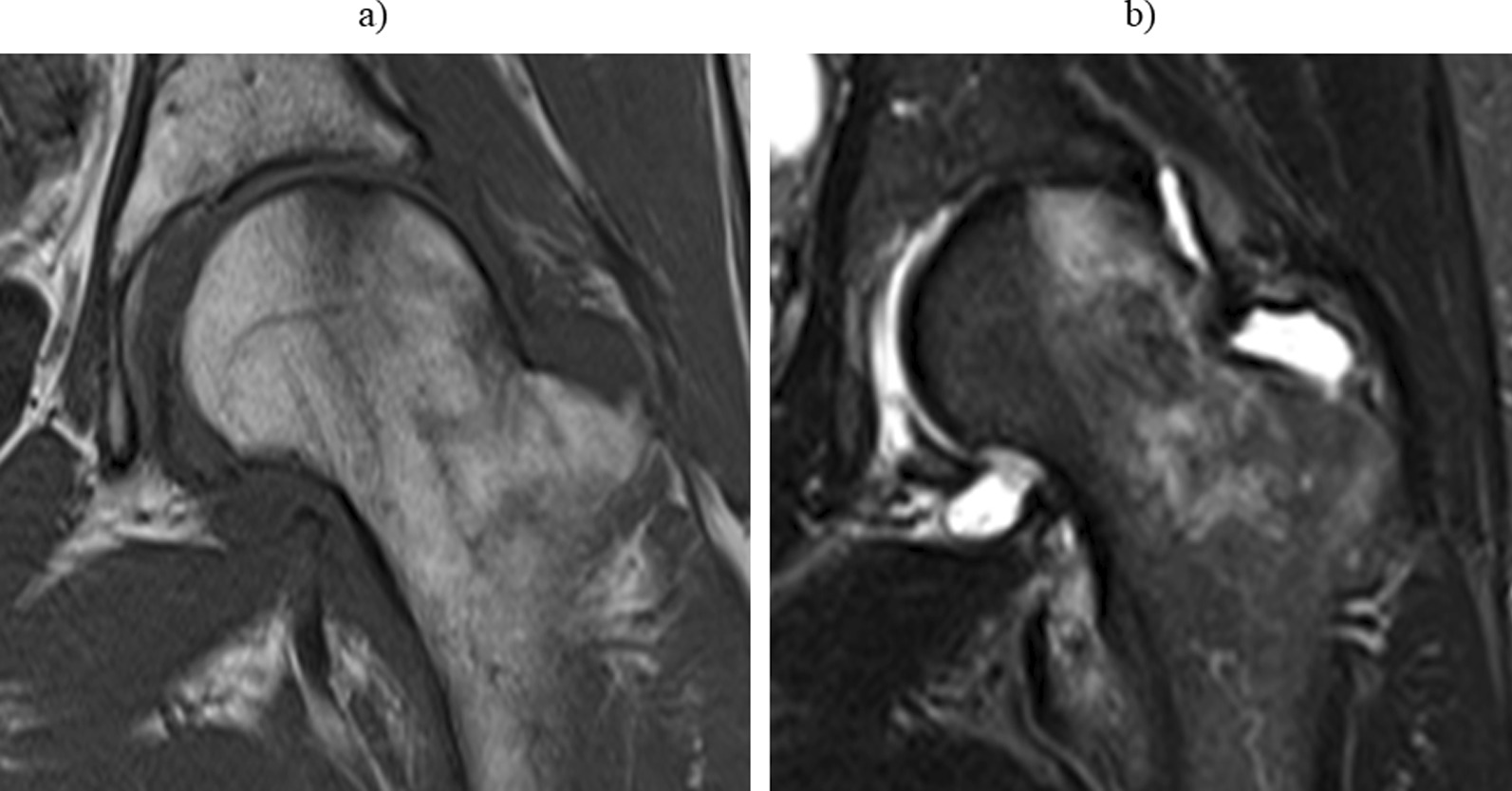
An example of bone marrow oedema (BME) in the femoral head. Magnetic resonance images of a middle-aged woman who was imaged 2 months after left hip pain without an apparent trigger. BME shows low signal intensity on a coronal T1-weighted image (**a**) and high signal intensity on a coronal short tau inversion recovery image (**b**). On measurement, the size of the BME was 3.2 cm. *BME* Bone marrow oedema

We determined the presence of BME on coronal short tau inversion recovery (STIR) and T1-weighted MR images. BME was graded by size (width of lesion, measured perpendicular to the adjacent articular surface) in three groups (no BME, BME ≤ 1 cm, BME > 1 cm) at any slice, as described by Sowers et al. [20]. An MRI image of a case with BME is shown in Fig. 2. We examined the association between BME and patient characteristics. Furthermore, we collected data on surgical treatments, such as hip arthroscopy, within a one-year follow-up period to determine whether the presence of BME affected the course of therapy.

### Statistical analysis

Statistical analysis was performed using SPSS version 19.0 software (SPSS, IL, Chicago, USA). Continuous data were expressed as the mean and standard error of the mean unless otherwise noted, and categorical data were expressed as *n* (%). The relationship between age, and MHHS pain and total score and BME (0: no BME, 1: BME ≤ 1 cm, 2: BME > 1 cm) was compared using one-way analysis of variance and post hoc by Tukey–Kramer test. The association between sex, surgical treatment, and BME was compared using Pearson's chi-square test. A *p* value < 0.05 was considered significant.

## Results

The patient characteristics are summarized in Table 1. The K–L classification was grade 0 in 29 cases (34.5%) and grade 1 in 55 cases (65.5%). The average MHHS pain and total scores were 25.8 ± 0.7 points and 62.5 ± 1.4 points, respectively. During the follow-up period, 52 patients (61.9%) underwent surgical intervention, including 51 cases (60.7%) of hip arthroscopy and 1 case of periacetabular osteotomy (1.1%).

**Table 1:** Patient characteristics

	*n* = 84
Sex	Male: 31 (36.9%)
	Female: 53 (63.1%)
Age (years)	46.5 ± 1.6
K–L grade	0: 29 (34.5%)
	1: 55 (65.5%)
MHHS pain score	25.8 ± 0.7
MHHS total score	62.5 ± 1.4
Surgical intervention within 1 year	+: 52 (61.9%)
	−: 32 (38.1%)

*MHHS* Modified Harris hip score

### Assessment of bone mallow oedema

The BME data are summarized in Table 2. In total, BME in the hip joint was found in 29 patients (34.5%), and large BME (> 1 cm) was found in 18 cases (21.4%). There was complete consensus agreement regarding the categorization of BME between the two observers.

**Table 2:** Presence and size of BME

	*N* = 84
Non-BME	55 (65.5%)
BME ≦ 1 cm	11 (13.1%)
BME > 1 cm	18 (21.4%)

*BME* Bone marrow oedema

The relationships between BME and patient characteristics are summarized in Table 3. There was a significant difference between the groups with respect to sex (*p* = 0.006), and there was no significant difference in age (non-BME vs. BME ≤ 1 cm: *p* = 0.074, non-BME vs. BME > 1 cm: *p* = 0.195).

**Table 3:** Relationship between BME and patient characteristics

	Non-BME (*N* = 55)	BME ≤ 1 cm (*N* = 11)	BME > 1 cm (*N* = 18)	*p* value
Sex	Male: 27	Male: 1	Male: 3	0.006*
	Female: 28	Female: 10	Female: 15	
Age (years)	49.2 ± 1.8	38.9 ± 4.4	42.6 ± 3.4	
Surgery for hip joint	+: 23, −: 32	+: 4, −: 7	+: 4, −: 13	0.563

*(*p* < 0.05)

**Fig. 3:**
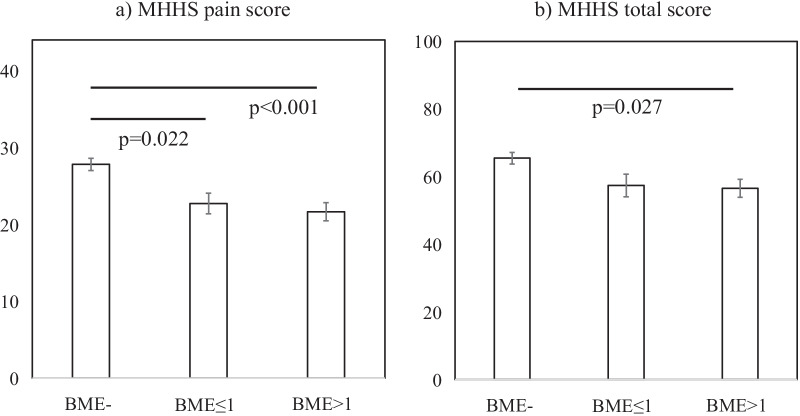
Relationships between BME and modified Harris hip score. The relationship between the three groups (non-BME, BME ≤ 1 cm, and BME > 1 cm) and **a** MHHS pain score, **b** MHHS total score were compared. A *p* value < 0.05 was considered significant. *BME* Bone marrow oedema, *MHHS* Modified Harris hip score

The MHHS pain score was significantly lower in the BME group than in the non-BME group, regardless of the BME size (non-BME vs. BME ≤ 1 cm: *p* = 0.022, non-BME vs. BME > 1 cm: *p* < 0.001, Fig. 3a). The MHHS total score was significantly lower in the BME > 1 cm group than in the non-BME group (non-BME vs. BME ≤ 1 cm: *p* = 0.131, non-BME vs. BME > 1 cm: *p* = 0.027, Fig. 3b). The presence of BME did not significantly differ between patients who did and did not undergo surgery within the one-year follow-up period (*p* = 0.563, Table 3).

## Discussion

In the present study, we examined the prevalence of BME in patients with LT, as well as its effect on the hip score and association with other factors. BME was found in 34.5% of patients with LT, with large BME (> 1 cm) in 21.4% of patients. The presence of BME in LT was significantly associated with lower MHHS scores (both pain and total scores).

There have been several reports on the frequency of BME, especially in the knee joint. In an MRI study of healthy individuals with radiographical knee OA and without knee pain, 39 of 297 individuals (13%) expressed BME in the knee joint [21]. Sowers et al. found that in cases of knee pain without obvious OA changes, 44.8% had knee BME and 8.6% had large BME (> 1 cm) [20]. Neumann et al. [17] described the frequency of BME in the hip joint; BME was found in 29% of patients with mechanical symptoms of the hip joint, such as pain, clicking, locking, and giving way. Kumar et al. reported patients with mild–moderate hip OA (KL2 or 3, *n* = 30) and healthy controls (KL0 or 1, *n* = 55). The results showed that 23.3% of patients with mild–moderate hip OA and 10.9% of controls had BME [22]. In the present study, the prevalence of BME in LT was comparable to previous reports for patients with hip pain or disease.

We also assessed the association between hip pain and BME in patients with LT. Regarding the knee joint, it has been reported that the prevalence of BME was higher in patients with symptomatic knee OA than in those without symptoms [23]. Additionally, large BMEs in knee OA have been reported to be associated with greater pain, which disappeared with resolution [24]. Furthermore, Sowers et al. clarified a significant association between BME > 1 cm and symptomatic knee OA [20]. Krammer et al. reported that the presence of BME and subchondral cysts in patients with hip OA were associated with a higher degree of pain and disability [22]. In this study, the presence of BME in LT was significantly associated with lower MHHS scores (both pain and total score). It was suggested that BME might contribute to hip pain and dysfunction in patients with LT.

In a recent review, BME was identified as an important cause of hip pain [25]. In a paper studying the pathogenesis of BME in mice with collagen-induced arthritis, BME was preceded by arthritic symptoms and synovitis at the onset of collagen-induced arthritis [26]. It was pointed out that after the appearance of BME, inflammatory signals related to the osteoclast environment, such as high expression of the nuclear factor κB ligand RANKL, increased cytokines and chemokines, and high activation of T cells and monocytes, occur in the bone marrow microenvironment, which may lead to synovitis and bone erosion. A study using histological analysis and microarray technology at sites of subchondral BME in the knee observed a high in situ turnover rate, pain sensitization, and activation of inflammatory signals [27]. Similar to the above-mentioned study, activation of inflammatory signals has been noted in humans as well. In the present study, BME was identified in patients with LT, and the pain scores were lower when BME was present. This suggested that BME might contribute to the pathogenesis of pain in patients with LT. To this end, BME may alter the bone marrow microenvironment into an osteoclastic environment, thus leading to the activation of inflammatory signals and causing pain.

Currently, conservative treatment for primary BME (bone marrow oedema syndrome) includes partial weight-bearing, immobilization, analgesics, anti-inflammatory drugs, and additional treatments such as external shock wave therapy, bisphosphonates, and iloprost [28–33]. Several randomized controlled trials have reported that bisphosphonate therapy significantly reduces BME size and pain in patients suffering from painful OA with BME [34, 35]. These point to the possibility that addressing the osteoclastic environment may have an effect. Moreover, although we were not able to examine it further in this study, we found differences between the groups with respect to gender. Further studies are needed to investigate pathomechanisms such as osteoporosis that may underlie the observed BME gender differences.

Several limitations of this study need to be recognized. First, a major limitation was the relatively small sample size due to the use of standardized data (i.e. same equipment and conditions). In addition, a lot of patients had to be excluded from the analysis. Second, we could not assess patient-reported pain outcomes (e.g. visual analogue scale) because during the study period we checked these only in preoperative patients. Despite these limitations, we believe that relevant and important information related to recognizing pain in patients with LT can be obtained from this study.

## Conclusion

BME was found in 34.5% of patients with LT. LT patients with BME had more pain than those without BME. BME on MRI in patients with LT might be an indicator of hip pain and hip joint dysfunction.

## Data Availability

The data sets supporting the conclusions of this article are included within the article. The raw data can be requested from the corresponding author.

## Abbreviations

LT: Hip labral tear
BME: Bone marrow oedema
MRI: Magnetic resonance imaging
STIR: Short tau inversion recovery
OA: Osteoarthritis
MHHS: Modified Harris hip score
K–L: Kellgren–Lawrence
RANKL: Nuclear factor κB ligand
